# Supplementary material for: Re-refinement of the structure of the planar hexagonal phase of ZnO nanocrystals
Source: Acta Crystallogr B Struct Sci Cryst Eng Mater. 2026 May 8;82(Pt 3):310–5. doi: 10.1107/S2052520626003860 (PMC13238490; doi:10.1107/S2052520626003860)
Supplement: Supplementary file 2 [file b-82-00310-sup2.pdf]

STRUCTURAL SCIENCE
CRYSTAL ENGINEERING
MATERIALS

Volume 82 (2026)

Supporting information for article:

Re-refinement of the structure of the planar hexagonal phase of ZnO nanocrystals

Musen Li, Lingyao Zhang, Wei Ren and Jeffrey R. Reimers

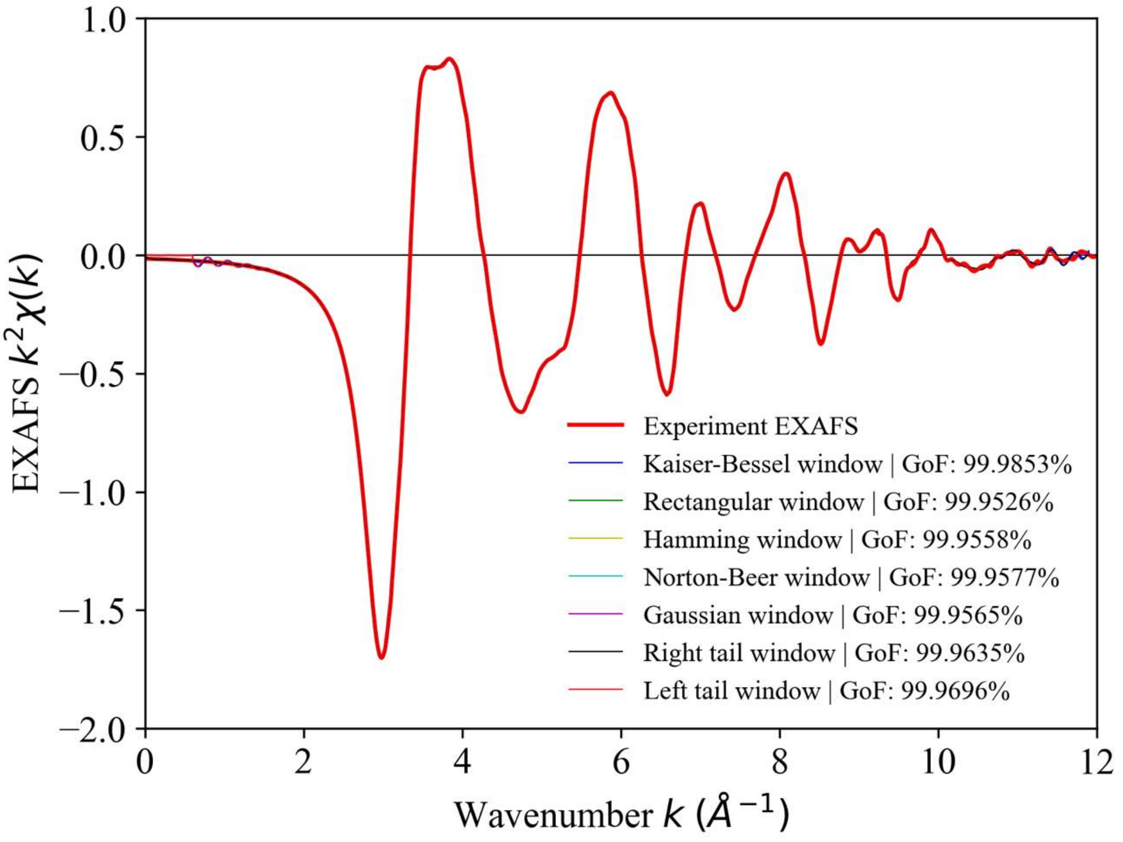

Figure S1 EXAFS spectrum showing the k^2-weighted $\chi(k)$ function plotted against wavenumber k ($Å^{-1}$) in the range of 0-12 $Å^{-1}$. The red curve represents experimental EXAFS data, and the colored curves represent calculated results that are all barely distinguishable from it. The goodness of fit (GoF) of backward FT EXAFSs using seven different window functions for Fourier transformation are listed: Kaiser-Bessel window (GoF: 99.9853%), Rectangular window (GoF: 99.9526%), Hamming window (GoF: 99.9558%), Norton-Beer window (GoF: 99.9577%), Gaussian window (GoF: 99.9565%), Right tail window (GoF: 99.9635%), and Left tail window (GoF: 99.9696%).

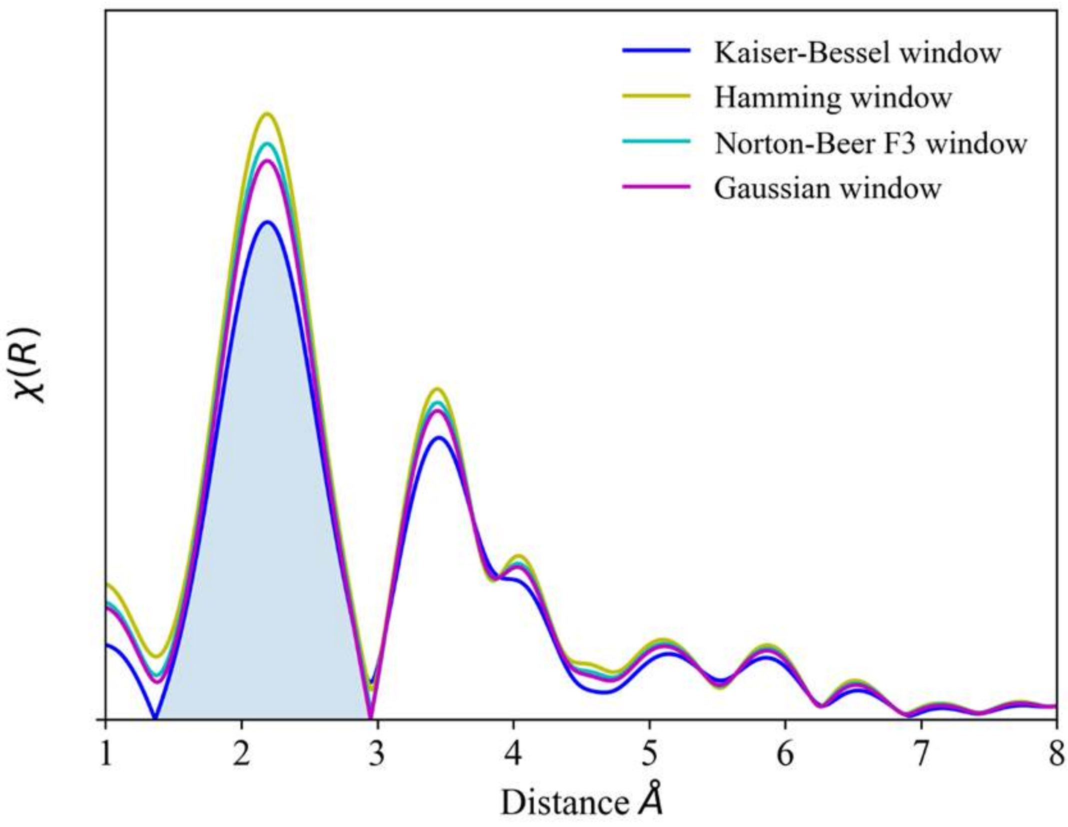

Figure S2 Pair distribution functions derived from EXAFS data using four different windowing functions: Kaiser-Bessel, Hamming, Norton-Beer F3, and Gaussian. Integration of the first peak area (shaded) shows that the Kaiser-Bessel window produces a coordination number of 5.03 ± 0.1. This is close to the theoretical value of 5 for h-ZnO, demonstrating optimal balance between spectral leakage suppression and signal fidelity.

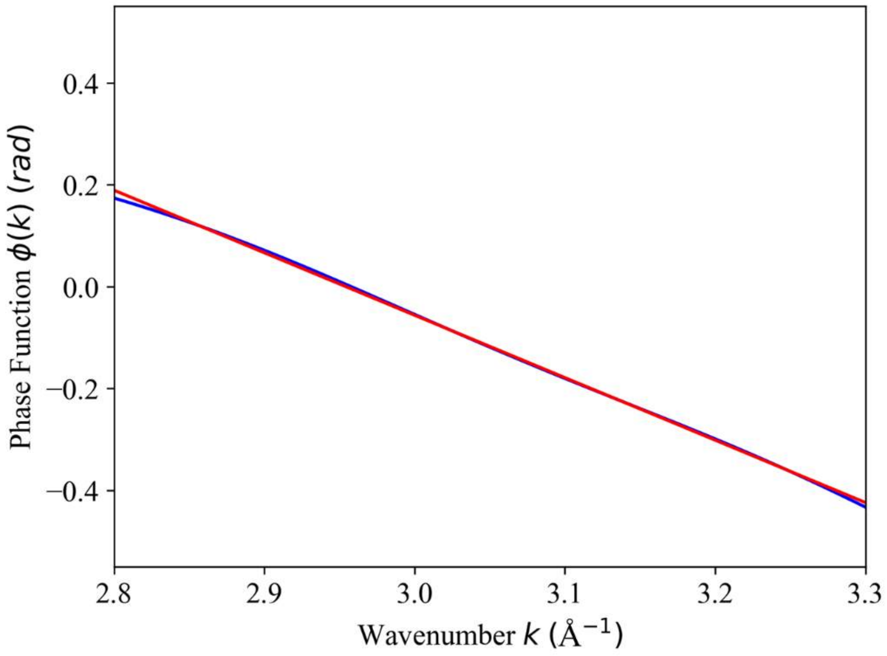

Figure S3 Figure S3 The phase function (Eqn. (1)) (blue) is plotted and fitted to a straight line (red) to extract the derivative $\frac{d\phi}{dk}$ for use in Eqn. (2).

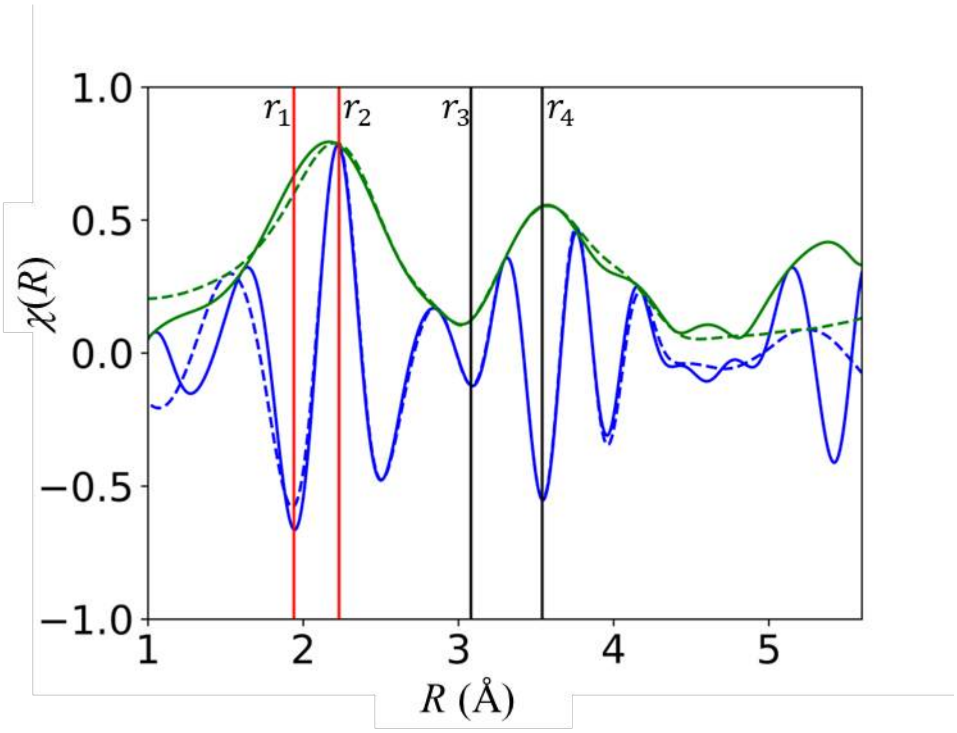

Figure S4 The PDF $\chi(R)$ obtained from the MD simulations performed at constant temperature and volume is used to determine thermal interatomic distances r_1 - r_4. This was obtained by Fiuriel-transformation of the EXAFS spectrum simulated from the simulation results using the method of Larch (Newville, 2013). Green- total PDF, blue- real part only; solid lines- MD simulation, dashed lines- observed from Fig. 3.
